# Droplet Microfluidics for Advanced Single‐Cell Analysis

**DOI:** 10.1002/smmd.70002

**Published:** 2025-04-04

**Authors:** Chang Liu, Xiaoyu Xu

**Affiliations:** ^1^ College of Chemistry and Material Science Shandong Agricultural University Taian China

**Keywords:** droplet microfluidics, genomic insights, high‐throughput screening, single‐cell analysis

## Abstract

Droplet microfluidics has emerged as a breakthrough technology that is changing our comprehension of single‐cell and their associated research. By separating individual cells within tiny droplets, ranging from nanoliters to picoliters using microfluidic devices, this innovative approach has revolutionized investigations at the single‐cell level. Each of these droplets serves as a distinct experimental reaction vessel, enabling thorough exploration of cellular phenotypic variations, interactions between cells or cell‐microorganisms as well as genomic insights. This review paper presents a comprehensive overview of the current state‐of‐the‐art in droplet microfluidics, which has made single‐cell analysis a practical approach for biological research. The review delves into the technological advancements in single‐cell encapsulation techniques within droplet microfluidics, elucidating their applications in high‐throughput single‐cell screening, intercellular and cell‐microorganism interactions, and genomic analysis. Furthermore, it discusses the advantages and constraints of droplet microfluidic technology, shedding light on critical factors such as throughput and versatile integration. Lastly, the paper outlines the potential avenues for future research in this rapidly evolving field.

## Graphical abstract

This paper reviews the transformative impact of droplet microfluidics on single‐cell research. It highlights current advancements in encapsulation techniques, applications in high‐throughput screening, and the study of intercellular and cell‐microorganism interactions. The discussion includes the advantages, limitations, and future research directions of this innovative technology in biological investigations.

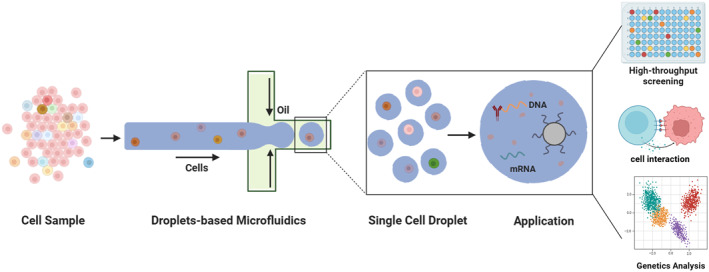

**Summary**

- Advances in methods for encapsulating individual cells in droplets enable single‐cell research.
- Applications in studying high‐throughput screening, intercellular interactions, and genomic analysis provide valuable insights.
- The review discusses the benefits of increased throughput and integration versus constraints affecting implementation.

## 1 Introduction

Cells represent the fundamental building blocks of biology, playing pivotal roles in structure and function. They encounter a myriad of factors that can impact their growth, specialization, and metabolic activities, resulting in notable variations among individual cells [1]. Consequently, scrutinizing the morphology and constituents of cells at the single‐cell level offers precise insights into the characteristics of individual cells within a specific microenvironment. This wealth of distinct profiles displayed by a multitude of individual cells is crucial for uncovering intercellular disparities, investigating cell signaling and the pathophysiology of ailments, and facilitating the identification of vital biomarkers for early disease detection [2]. Single‐cell studies are closely linked to crucial biological substances, encompassing nucleic acids, proteins, peptides, small molecule metabolites, and trace elements. These components play pivotal roles in a wide range of cellular functions and are essential for sustaining cellular vitality. Nevertheless, the detection and analysis of these substances and their intricate combinations within individual cells pose significant challenges owing to the limited sample volumes and the diverse molecular milieu within cytological environments [3]. To accurately characterize and ultimately unravel the underlying causes of cellular heterogeneity, a substantial number of single‐cells must be analyzed to adequately represent a given population. In recent decades, a multitude of high‐throughput analysis techniques have been employed in single‐cell assays [4]. These methods encompass single‐cell transcriptomics [5], flow cytometry [6], fluorescence microscopy [7], Raman spectroscopy [8], and electrochemical analysis [9]. However, these approaches suffer from various limitations including limited selectivity, poor repeatability, and low reliability. Moreover, many of these methods rely on cell labeling and are constrained by the quantity of molecules that can be analyzed, primarily due to the absence of robust single‐cell segmentation capabilities.

In recent years, microfluidics, especially droplet microfluidics, has emerged as an accessible, high‐throughput, and cost‐effective method for single‐cell analysis [10]. Microfluidics entails the manipulation of tiny droplets, ranging from nanoliters to picoliters, within micron‐scale channels [11], rendering it highly compatible with the size of individual cells. This feature makes it exceptionally suitable for conducting comprehensive biochemical and biophysical characterization of cells [12]. Within the realm of droplet microfluidics, the rapid mixing of immiscible phases gives rise to droplets, a process that typically occurs at microfluidic junctions, where immiscible fluids are forced to interact under controlled pressure and flow conditions, paving the way for a myriad of applications including directed evolution, tissue printing, polymerase chain reaction (PCR), and beyond [13]. It also holds significant promise for single‐cell analysis, where individual cells can be confined within their own encapsulation environments. This isolation allows for the accumulation of secreted molecules, facilitating the attainment of assayable concentrations for precise analysis [14].

**FIGURE 1:**
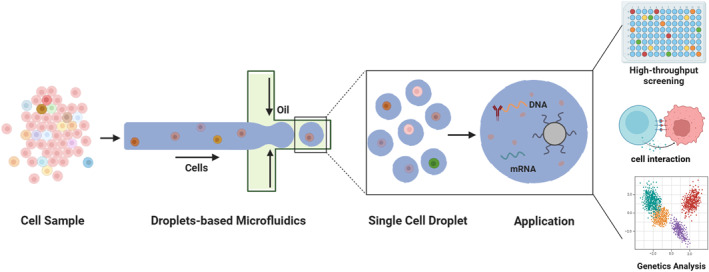
An overview of the single‐cell droplet microfluidics for advanced biological applications.

The objective of this review is to offer a succinct yet comprehensive overview of the remarkable advancements achieved through the application of droplet microfluidics in single‐cell analysis (Figure 1). Structured meticulously, the review begins by providing an introduction to droplet microfluidics, elucidating its key attributes that make it an ideal technique for single‐cell analysis. Subsequently, the review takes an in‐depth look at the areas of technological advancements in capturing single‐cell in droplets, delineating the innovative strategies employed to enhance this process. Moving forward, the review transitions to an exploration of recent breakthroughs in single‐cell epigenetics, revealing the intricacies of cellular regulation and gene expression patterns at the individual cell level. Additionally, it examines the burgeoning field of cell‐cell and cell‐microbe interactions, unraveling the complexities of intercellular communication and symbiotic relationships. In addition, this review ventures into the arena of single‐cell genomics, providing insights into unveiling the genetic blueprint of individual cells, thus paving the way for a deeper understanding of cellular heterogeneity and function.

## 2 Droplet Microfluidics Technology

**FIGURE 2:**
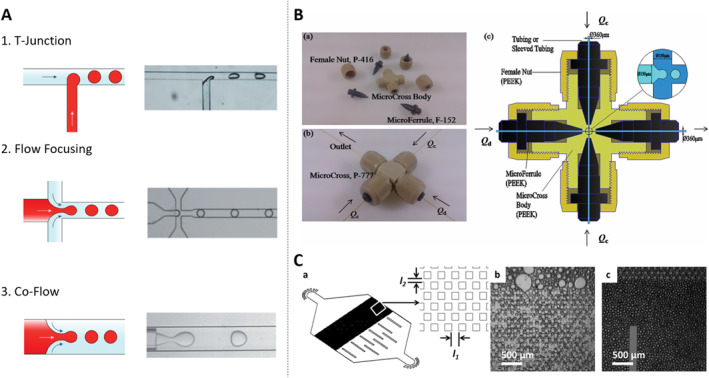
Droplet microfluidics generation geometries applied in single‐cell workflows. (A) Three kinds of channel structure to generate droplets in microfluidic systems. Reproduced with permission [18]. Copyright 2020, John Wiley and Sons. (B) Scheme of the micro‐cross and its detailed structure and internal geometrical dimensions. Reproduced with permission [19]. Copyright 2014, Royal Society of Chemistry. (C) Schematic illustration of the post‐array device. Reproduced with permission [20]. Copyright 2014, Royal Society of Chemistry.

Droplet microfluidics is founded on the principle of utilizing two or more immiscible liquids, each designated as either the dispersed phase or the continuous phase. Within this setup, the dispersed phase is fragmented into minuscule volumes within the continuous phase. Droplets form spontaneously at the interface of these two phases driven by the combined forces of surface tension and shear force. This phenomenon, initially observed in capillaries, was later successfully adapted for application in microfluidic channels, a pioneering innovation attributed to the Weitz team [15]. In the realm of droplet microfluidics systems, three primary channel structures are commonly employed for droplet generation: the T‐junction structure, flow focusing structure, and co‐flow structure (Figure 2A) [16]. The classification of droplets into two types, W/O (water‐in‐oil) and O/W (oil‐in‐water), hinges on the wettability of the inner channel wall and the distinctions between the dispersed and continuous phases. Furthermore, multiple emulsions represent intricate polydisperse systems where both O/W and W/O emulsions co‐exist simultaneously. These emulsions are stabilized by hydrophobic and hydrophilic surfactants, respectively. The capability to conduct millions of independent experiments concurrently with exceptional reproducibility in a short timeframe has had a profound impact on the advancement of high‐throughput screening technology. This innovation has significantly accelerated research endeavors, particularly in fields such as drug discovery, where the ability to rapidly screen numerous compounds or conditions is paramount. Besides, aqueous two‐phase systems (ATPS) have emerged as an innovative strategy beyond conventional O/W or W/O systems, particularly for biological applications [17]. ATPS‐based droplets offer distinct advantages, such as enhanced biocompatibility, selective biomolecule partitioning, and tunable phase separation properties, making them highly suitable for single‐cell analysis and biomolecular assays. Incorporating ATPS within droplet microfluidics expands the range of applications, including cell encapsulation, protein crystallization, and enzymatic reactions, under physiologically relevant conditions. Moreover, the versatility and scalability of droplet microfluidics systems offer promise across various domains including biotechnology, diagnostics, and materials science. Through continued innovation and refinement, droplet microfluidics continues to emerge as a cornerstone technology enabling unprecedented advancements in diverse scientific disciplines.

In the domain of droplet generation technology, Wu et al. introduced an innovative and easy‐to‐assemble 3D Micro‐Cross droplet generation structure utilizing connectors (Figure 2B) [19]. This approach presents several advantages over conventional methods for generating droplets. First and foremost, the Micro‐Cross structure seamlessly integrates with the droplet detection device, providing a solution to the challenge of droplet merging. This means that the generated droplets can be effortlessly collected and processed within a capillary tube, thereby eliminating the need to address droplet coalescence issues. Secondly, unlike traditional methods that necessitate consideration of the hydrophilicity and hydrophobicity of the chip material, the Micro‐Cross structure can generate both W/O and O/W droplets without requiring any special wettability treatment on the inner wall of the device. This simplifies the manufacturing process and enhances flexibility. Lastly, the three‐dimensional design of the Micro‐Cross structure allows droplets to be separated in either direction, offering greater versatility. Additionally, the droplet generation frequency achieved with this approach surpasses that of conventional two‐dimensional polydimethylsiloxane (PDMS) chips, making it a valuable advancement in the field of droplet microfluidics.

Weitz’s group has pioneered the development of an innovative microfluidic device featuring a distinctive micropillar array structure. This design facilitates the sequential splitting of larger emulsion droplets, thereby yielding smaller and more uniformly dispersed droplets, as illustrated in Figure 2C [20]. Unlike traditional methods of droplet generation, this novel structure exhibits minimal sensitivity to the viscosity of the working fluid. Consequently, it can be effectively utilized with a wide range of fluids, regardless of their varying viscosities. Additionally, this microfluidic device boasts enhanced droplet generation capabilities, achieving a remarkable production rate of approximately 250 L/h/m^2^. Moreover, it enables precise control over droplet size, significantly reducing variation to just 13% compared to the conventional method's 23%. The droplets produced by this innovative device are distinguished by their uniformity and speed, rendering them an appealing option for applications in industrial production. When the dispersed phase comprises a dilute cell suspension, these droplets facilitate the encapsulation of individual cells, thereby unlocking a spectrum of possibilities including merging, splitting, incubation, assays, and sorting. A notable advantage of this technology lies in its capacity to generate droplets in large quantities, effectively surmounting the limitations associated with conventional methods such as well plates. In addition to these primary droplet microfluidics strategies, other approaches such as centrifugal droplet generation method, digital microfluidics, and membrane emulsification can provide additional insights and comprehensive understanding of the technology. The centrifugal droplet generation method leverages the forces generated by the rotation of a microfluidic device to create droplets [21]. This method offers advantages in terms of droplet uniformity and control over droplet size, making it a valuable alternative to traditional droplet generation techniques. Digital microfluidics, on the other hand, involves the manipulation of droplets on an array of electrodes [22]. By applying voltage to specific electrodes, droplets can be moved, merged, split, and otherwise manipulated with high precision. This technique is particularly well‐suited for applications requiring complex droplet handling and reaction sequences. Membrane emulsification utilizes a porous membrane to generate droplets [23], which is known for its ability to produce droplets with a narrow size distribution and high encapsulation efficiency. It is particularly useful in applications where the encapsulation of cells or other particles within droplets is critical. In the subsequent sections, we will delve into the evolution of the single‐cell droplet microfluidics technique and explore how it can be harnessed to extract valuable insights from extensive populations of single‐cell.

## 3 Single‐Cell Encapsulation in Droplet Microfluidics

### 3.1 Single‐Cell Encapsulation

The study of encapsulating live cells has been the subject of extensive research over the past decades. During this time, a wide variety of microfluidic materials and devices have been created to facilitate the encapsulation of live cells within droplets. The quantity of cells contained in each individual droplet can be estimated by employing a Poisson distribution, which provides the probability, denoted as P(*X* = *k*), of finding exactly *k* cells per droplet and is described by the following equation:

(1) $$f\left(k;\lambda \right)=\mathrm{Pr}\left(X=k\right)=\frac{\lambda^{k}e^{-\lambda }}{k!}$$

*λ* denotes the average number of cells in each droplet volume. As can be seen from Equation (1), the average count of cells in a droplet is varied by adjusting the concentration of cells in the aqueous phase. For example, in order to wrap *λ* = 0.3 cells in a 50 pL droplet, a cell concentration of 6 × 10^6^ cells/mL is required. Considering that the procedure of cell wrapping is utterly random, this selection of cell concentration would lead to 74.08% of the droplets without cells, 22.22% containing single‐cell, 3.3% with two cells, and 0.38% containing more than two cells.

**FIGURE 3:**
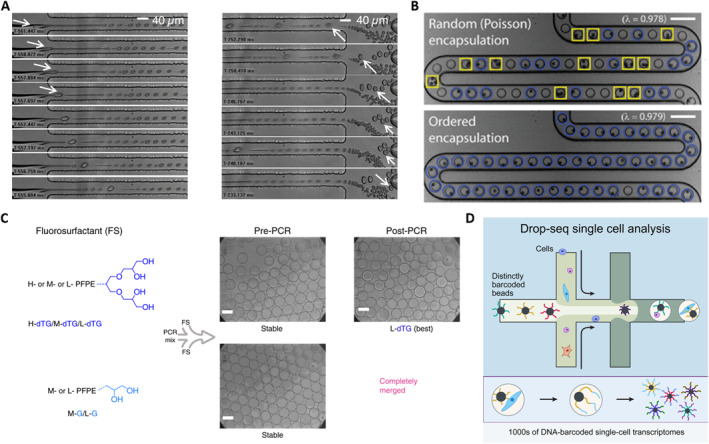
Droplet microfluidic‐based single‐cell encapsulation, droplet stability, and labeling for single‐cell analysis. (A) The process of cell encapsulation where (left) cells cause jet neck breakage and destabilization, and (right) the sorting process where smaller positive droplets are pushed down by larger droplets. Reproduced with permission [24]. Copyright 2008, National Academy of Sciences. (B) Inertial flow‐based cell spacing and single‐cell encapsulation using a high aspect‐ratio microchannel with the stochastic and ordered encapsulation. Reproduced with permission [25]. Copyright 2008, Royal Society of Chemistry. (C) Polar head group geometry dictates droplet stability. Reproduced under terms of the CC‐BY license [26]. Copyright 2019, The Authors, published by Springer Nature. (D) Highly parallel genome‐wide expression profiling of individual cells using nanoliter droplets. Reproduced with permission [27]. Copyright 2015, Elsevier.

To address the limitations of the Poisson distribution and enhance the encapsulation rate of single‐cell, Viovy et al. proposed a simple hydrodynamic approach [24]. In their method, after each cell enters the narrow water jet formed in the flow focusing device, the droplets containing single‐cells spontaneously self‐sort from smaller empty droplets, inducing their own encapsulation. The encapsulation progress relies on the Rayleigh‐Plateau instability triggered by cells in the flow focusing geometry as illustrated in Figure 3A. Additionally, self‐sorting is dependent on two hydrodynamic mechanisms: lateral drift of deformable objects in a shear flow and sterically driven dispersion in a compressional flow. When the cell suspension is employed as the aqueous phase, the cells access the focusing zone causing the jet “neck” to rupture. This smart mechanism can also be utilized for cell sorting as the droplet size is always slightly larger than the size of the cells they contained. It has been reported that 70%–80% of droplets containing only one cell are successfully encapsulated and sorted, indicating a considerable improvement over random cell encapsulation. In addition, the passive sorting of droplet microfluidics by their size is also achieved through deterministic lateral displacement. In this method, an inclined columnar array allows droplets smaller than a specific critical diameter to flow in the direction of the inflowing liquid, while larger droplets are confined in the inclined channels of the columnar array [28]. Using this microfluidic construction, recent investigations have successfully separated droplets containing shrunken yeast cells from 31% of the large‐diameter droplets containing only culture medium, as well as separating large droplets encasing tumor cells from empty small droplets, resulting in an enrichment of single‐cell encapsulated droplets to 78%.

Meanwhile, inertial focusing has emerged as a promising technique to enhance the encapsulation efficiency of single‐cells in droplet microfluidics. Toner et al. proposed a new method to self‐organize cells into two uniformly spaced streams before the emulsification process (Figure 3B) [25]. This approach involves forcing a highly concentrated suspension of cells or particles rapidly through a microchannel with a high aspect ratio. As the suspension moves longitudinally over half the distance of the particle‐particle spacing, the particle diameter occupies a significant portion of the narrow dimensions of the channel. This passive approach effectively overcomes the inherent limitations of Poisson's statistical theory and greatly improves the wrapping rate of single‐cell within droplets. Additionally, Berg's research team introduced a novel method to address the challenge of low single‐cell encapsulation rate by leveraging a second force known as the Dean force [29]. This force, induced by curvature, focuses the particles to a single equilibrium position generated by the presence of secondary flow resulting from the difference between the downstream velocity of the central fluid and the fluid near the channel wall [30]. This inertial sorting technique in curved microchannels effectively sorts cells leading to an increase in the wrapping rate of single‐cell to 77%. Furthermore, the droplet generation rate using this method can reach up to 3500/s, demonstrating its efficiency and speed in encapsulating single‐cells. While efforts have been made to address the challenge of low efficacy in individual cell encapsulation, with optimized efficiency reaching around 80% at best, there remains room for improvement. It is recommended that further research be conducted to advanced technology development in this area. However, when considering the issue from an application point of view, it becomes debatable whether there is a need to further increase the efficiency of single‐cell encapsulation.

### 3.2 Droplet Stability

Single‐cell encapsulation with the stability of droplets remains a critical focus for advancing both fundamental research and practical applications. The fluorocarbon oils are highly suitable for droplet‐based explorations due to their excellent solubility for dissolved oxygen, which is essential for the viability of encapsulated cells. However, utilizations such as PCR and drug screening are limited by droplet instability during thermal cycling, as coalescence often occurs at temperatures above 80°C, compromising reliability. Surfactants play a crucial role in stabilizing water‐in‐fluorocarbon emulsions, preventing droplet merge, and ensuring a biologically inert interior surface. Among available options, fluorosurfactants with long fluorocarbon tails, such as perfluorinated polyethers (PFPE), provide robust stabilization but can impair the activity of encapsulated biomolecules due to ionic head groups. To address this, Weitz et al. developed a non‐ionic fluorosurfactants that combine PFPE tails for droplet stability and polyethylene glycol (PEG) headgroups to prevent biomolecule adsorption [31]. These surfactants successfully stabilized emulsions, maintained biocompatibility, and supported applications like in vitro translation (IVT) and yeast cell growth. However, challenges persisted, including droplet merging during thermal cycling and inter‐droplet transfer of small organic molecules, attributed to limitations in surfactant structure and synthesis. In 2019, Weitz’s group advanced surfactant design with dendronized fluorosurfactants incorporating mono‐ or tri‐glycerol polar head groups and fluoro‐tails of varying lengths [26]. The best‐performing surfactants featured four hydroxy groups in their polar head which enhanced hydrogen bonding providing improved high‐temperature stability and small molecule retention compared to PEG‐PFPE surfactants (Figure 3C). These hydroxy groups also offered functionalization opportunities for specific applications. The new surfactants exhibit superior performance in droplet‐based PCR and chemical retention, making them highly suitable for experiments requiring robust droplet integrity and compatibility with encapsulated biomolecules.

### 3.3 Droplet Labeling

In droplet microfluidics, when multiple reagents are introduced, each droplet can function as an effective microreactor if the contents of the millions of generated droplets are identifiable. For example, in research fields like high‐throughput screening, which heavily relies on droplet microfluidics, identical sample droplets (e.g., single‐cell droplets) are continuously and extensively paired with testing droplets (often referred to as droplet libraries). The diverse contents encapsulated in the testing droplets correspond to different reaction conditions, serving as carriers of experimental variable information throughout the analysis process. Thus, in systems involving multiple reagents, the labeling and indexing of droplets are critical for tracking their contents. There are three common methods for droplet labeling: positional, fluorescent and oligonucleotide sequence encoding.

Positional encoding relies on the position or arrangement order of the droplets as a means to identify their contents. While positional encoding is the simplest approach among droplet labeling techniques, it does not require additional encoding reagents within the droplets. This advantage makes it suitable for high‐throughput screening applications as it eliminates the potential adverse effects of encoding reagents on cells. Weitz et al. described a droplet‐based microfluidic system for analyzing DNA molecules dispersed in a solution. To utilize this system for DNA sequence analysis, the solution is injected into droplets containing probes via picoinjection [32]. They demonstrated a robust technique that uses electro‐microfluidics to add reagents to individual droplets. By employing a pressurized channel, a controlled volume of reagent is injected into each droplet, triggered by an electric field. This approach is analogous to a microarray, where each position on the array tests for a specific 15‐base sequence. However, instead of performing reactions on a physical array, their system conducts them in sequential sets of flowing microdroplets. To identify droplet contents, a labeling scheme using fluorescent dyes was developed, enabling affordable and high‐throughput DNA sequence analysis.

Fluorescent encoding involves adding appropriate concentration of fluorescent reagents to the dispersed phase solution before generating testing droplets. The contents of the droplets are then decoded by detecting different fluorescence wavelengths and intensities. Weitz et al. incorporated fluorescent reagents into single‐cell droplets to detect enzymatic activity on the cell surface through fluorescence emission. Furthermore, the uniform droplet size ensures accurate comparison of fluorescence intensities, enabling precise measurement of enzymatic reaction rates. Using this strategy, the system was applied to directed evolution, identifying new mutants of the enzyme horseradish peroxidase with catalytic rates over 10 times faster than their already highly efficient parent enzyme. The system leverages ultrahigh throughput to perform an initial purifying selection, eliminating inactive mutants and identifying approximately 100 variants with activity comparable to the parent enzyme from an initial population of about 10^7^. After a second round of mutagenesis and high‐stringency screening, several significantly improved mutants were identified, some approaching diffusion‐limited efficiency. However, this method has notable drawbacks. It heavily relies on external equipment such as expensive fluorescence microscopes and optical filters. Additionally, the instability of fluorescent reagents presents a potential limitation for droplet labeling via fluorescent encoding.

Using DNA oligonucleotide sequences enables the encoding of a larger number of samples, with encoding capacity corresponding to 4^n^ (where *n* is the length of the nucleotide sequence). This capacity is virtually unlimited, making DNA oligonucleotide sequences, also known as DNA barcodes, a powerful tool for droplet labeling. Weitz et al. reported a platform that combines droplet microfluidics with DNA barcoding to achieve genome‐wide expression profiling at single‐cell resolution [27]. This approach, known as Drop‐seq (Figure 3D), allows for rapid profiling of thousands of individual cells by encapsulating them into nanoliter‐sized aqueous droplets, associating a unique barcode with each cell's RNA, and performing pooled sequencing. Drop‐seq can simultaneously analyze mRNA transcripts from thousands of individual cells while retaining the information on each transcript's cell of origin. For instance, transcriptomes from a large number of mouse retinal cells were analyzed, revealing multiple transcriptionally distinct cell populations and constructing a molecular atlas of gene expression for known retinal cell classes as well as novel candidate cell subtypes. Drop‐seq represents a significant advancement in biological research, facilitating routine transcriptional profiling at single‐cell resolution and accelerating discoveries in the field.

## 4 The Integrated Droplet Microfluidics for Single‐Cell High Throughput Screening

Droplet microfluidics devices have been developed and integrated to explore biological questions, which are adept at high‐throughput screening. These devices enable encapsulation of individual cells within droplets, coupled with mutation‐specific PCR or other DNA/RNA amplification techniques, facilitating rapid screening of millions of cells for the presence of the mutation of interest. This approach has proven invaluable in swiftly identifying rare mutations in tumor cells, aiding in the development of personalized therapies [33]. Additionally, Weitz's methodology extends beyond genome screening to include phenotypic screening for desired traits, such as identifying cells producing specific antibodies or as a key component in an automated system for enzyme evolution.

**FIGURE 4:**
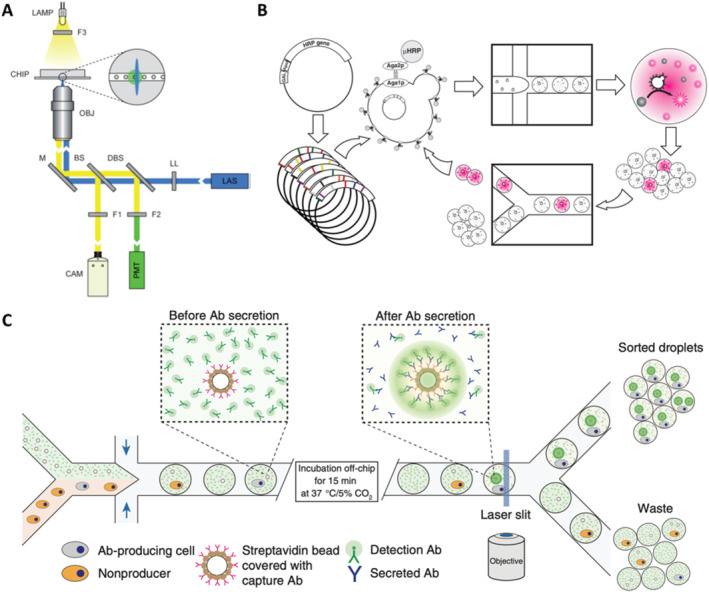
Integrated droplet microfluidics system for single‐cell high throughput screening. (A) The optical setup uses a laser to excite droplets in a microfluidic chip, detecting fluorescence with a photomultiplier tube. Reproduced with permission [34]. Copyright 2009, Royal Society of Chemistry. (B) The directed evolution experiment involves transforming yeast with HRP gene libraries, coencapsulating yeast and substrate in droplets for screening, sorting the brightest mutants based on fluorescence, and iterating the growth and sorting process. Reproduced with permission [35]. Copyright 2010, National Academy of Sciences. (C) The experimental design involves mixing two cell types in a microfluidic device to co‐encapsulate antibody‐producing cells with fluorescent beads, enabling sorting of droplets that contain fluorescent beads linked to antibody‐producing cells after incubation. Reproduced with permission [36]. Copyright 2013, Springer Nature.

The droplet microfluidics system developed for single‐cell screening integrates the pivotal elements of cell packaging, incubation, fluorescence detection of metabolites as well as droplet sorting by fluorescence intensity. This innovative system has been successfully applied as a robust platform for screening β‐galactosidase activity of E. coli. For this purpose, Weitz et al. have presented a droplet microfluidics system that performs fluorescence‐activated cell sorting according to the enzymatic activity of packaged single‐cells [34]. The mixture of E. coli cells expressing the reporter enzyme β‐galactosidase or an inactive variant is isolated from the fluorescent substrate and further sorted in a fluorescence‐activated manner shown in Figure 4A. This sorting process achieves a throughput of up to 300/s, with a false positive error rate of less than 1 in 10^4^ droplets, highlighting its efficiency and reliability. The findings indicate that the primary limitation to enrichment lies in the encapsulation of E. coli rather than sorting errors. Notably, the higher sorting efficiency is observed with the lower density of cell encapsulation, ensuring that all recovered cells are active strains. Building upon this success, they introduced a universal ultra‐high throughput screening platform, dramatically scaling up the screening process [35]. This platform enables the screening of thousands of droplets dispersed in oil phase as picoliter‐volume reaction vessels every second. To validate the effectiveness of our approach, they applied the assay to directed evolution and successfully identified a new mutant of horseradish peroxidase exhibiting more than a tenfold increase in catalytic activity compared to its parent enzyme, already renowned for its efficiency as Figure 4B. Leveraging initial purification selection to eliminate inactive mutants, they identified 100 mutants with activity levels comparable to the parent from an initial pool of 10^7^ mutants. Subsequent rounds of mutagenesis and highly stringent screening led to the identification of several markedly improved mutants, some of which are already close to the efficiency of the diffusion limit. Overall, they screened 10^8^ individual enzymatic reactions within a mere 10‐h timeframe, utilizing less than 150 μL of total reagents. This represents a staggering improvement of 1000‐fold in speed and at a million‐fold reduction in cost compared to state‐of‐the‐art robotic screening systems, underscoring the unprecedented efficiency and cost‐effectiveness of our approach.

Isolation of individual cells within droplets makes it possible to analysis the proteins secreted by these cells, thus overcoming the main limitations of conventional flow cytometry and fluorescence‐activated cell sorting. This method has been employed to screen individual mouse hybridoma cells for their antibody secretory capacity (Figure 4C) [36]. In this technique, individual mouse hybridoma cells, along with fluorescent probes and single beads labeled with anti‐mouse IgG antibody, are encapsulated in 50 pL droplets. As the microbeads capture the secreted antibody and subsequently bind to the fluorescent probe, the fluorescence becomes localized on the microbeads, generating a distinct and identifiable fluorescent signal for droplet sorting up to 200 Hz, enabling cell enrichment. The presented droplet microfluidics system is highly adaptable for screening various intracellular, cell surface or secreted proteins as well as quantifying the catalytic or regulatory activities. The phenotyping of single‐cells based on substances they secrete or consume is critical in numerous biological applications, such as combinatorial metabolic engineering for the overproduction of secreted metabolites. For instance, Weitz et al. successfully isolated *Saccharomyces cerevisiae* cells exhibiting high xylose depletion from a population with a frequency of one such cell per 10^4^ cells by droplet microfluidics. Subsequently, screening a genomic library led to the identification of multiple copies of the xylose isomerase gene as a genomic variant responsible for the high xylose depletion trait, which is significant for lignocellulosic feedstock utilization. Similarly, they achieved a 5800‐fold enrichment of L‐lactate producing E. coli clones from a population with a frequency of one L‐lactate producer per 10^4^ D‐lactate producers [37]. High throughput screening of biological phenotypes is essential for better understanding the genotype‐to‐phenotype linkage in living systems. Single‐cell microfluidic assays are used to characterize the enzymatic activity of millions of single‐cells, creating large‐scale activity‐to‐genotype mappings for machine learning approaches [38]. Additionally, they are developing single‐cell continuous directed evolution approaches to harness new genetics and high‐throughput screening for enhancing antibodies and enzymes. This integrated approach holds immense potential for advancing our understanding of biological systems and facilitating the development of improved biotechnological solutions.

## 5 Droplet Microfluidics for Cell‐Cell or Cell‐Microbe Interaction Analysis

Droplet microfluidics has shown immense potential in biological research especially in studying cell‐cell or cell‐microbe interactions. By encapsulating multiple types of cells or microorganisms within individual droplets, researchers can precisely observe their interactions. For example, bacteria and mammalian cells can be encapsulated together to deeply analyze how pathogenic and non‐pathogenic bacteria interact with mammalian cells [39]. This method overcomes the limitations of traditional approaches allowing for a more detailed study of dynamic cellular interactions.

Droplet microfluidics is not only applied to the study of bacteria and mammalian cells but also to the investigation of interaction mechanisms between individual viruses and mammalian cells. Compared to conventional well plate methods, droplet microfluidics can conduct up to a thousand times more cell interaction experiments within the same time frame, significantly enhancing experimental throughput and data acquisition efficiency. Quantitative analysis of cytokine secretion is critical for characterizing immune responses, particularly in studying functional aspects of immune effector cells such as natural killer (NK) cells. Traditional cytokine capture assays typically use engineered antibodies to immobilize secreted cytokines on the secreting cells, allowing sensitive identification and recovery of these cells. However, this approach can result in misidentification if cytokines diffuse, causing non‐secreting cells to be mistakenly recognized as responding cells.

**FIGURE 5:**
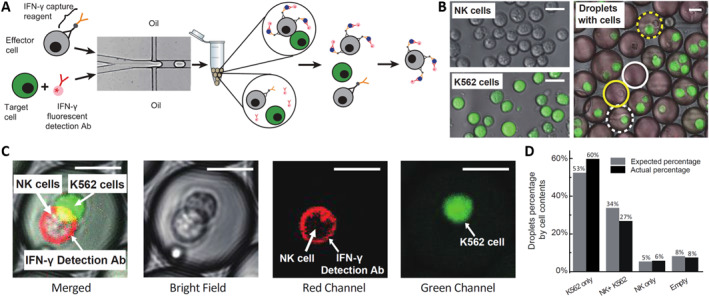
Droplet microfluidics encapsulation for detecting cell–cell or cell–microbe interaction. (A) Co‐encapsulation of effector and target cells in droplets for activation and sorting. (B) Measurement of encapsulation efficiency using labeled effector and target cells. (C) Detection of IFN‐*γ* secretion from activated effector cells in co‐encapsulation. (D) Comparison of actual and predicted cell distribution in droplets. Reproduced with permission [40]. Copyright 2020, Royal Society of Chemistry.

To address this issue, researchers encapsulate immune cells within droplets and perform cytokine capture assays inside the droplets, thereby restricting the diffusion of secreted cytokines in Figure 5. Specifically, NK‐92 MI cells and their target K562 cells are rapidly encapsulated within droplet microfluidics [40], where intra‐droplet IFN‐γ capture assays are conducted. This demonstrates that NK‐92 MI cells recognize the target cells within droplets and are activated to secrete IFN‐γ. Droplet encapsulation effectively prevents secretions from spreading to neighboring cells, significantly reducing false positives and negatives compared to assays without droplets. Once the cells are released from the droplets, the secreted cytokines remain attached to the secreting immune cells, facilitating the isolation of a highly enriched population of activated effector immune cells through fluorescence‐activated cell sorting (FACS). This encapsulation technique provides a novel approach to improve detection accuracy and reliability in single‐cell secretion assays.

Rapid and accurate identification of pathogenic bacteria in environmental samples is crucial for clinical diagnostics and public health. Approximately 80% of foodborne illnesses are caused by “unknown sources,” primarily due to the low abundance of pathogens in samples or a lack of targeted detection methods. Traditional bacterial culture and detection methods are often time‐consuming and require prior knowledge of pathogens, limiting their practical utility. To overcome these challenges, droplet microfluidics is used to develop high‐throughput pathogen screening methods that do not rely on prior pathogen knowledge. Weitz et al. encapsulated human tissue and target bacteria within droplets, forming a functional assay platform capable of screening up to 10^6^ bacteria per day [41]. This innovative approach allows rapid assessment of bacterial pathogenicity and efficient capture of low‐abundance pathogens in samples, greatly enhancing the detection rate and diagnostic accuracy of foodborne diseases.

## 6 Droplet Microfluidics for Single‐Cell Genomic Analysis

**FIGURE 6:**
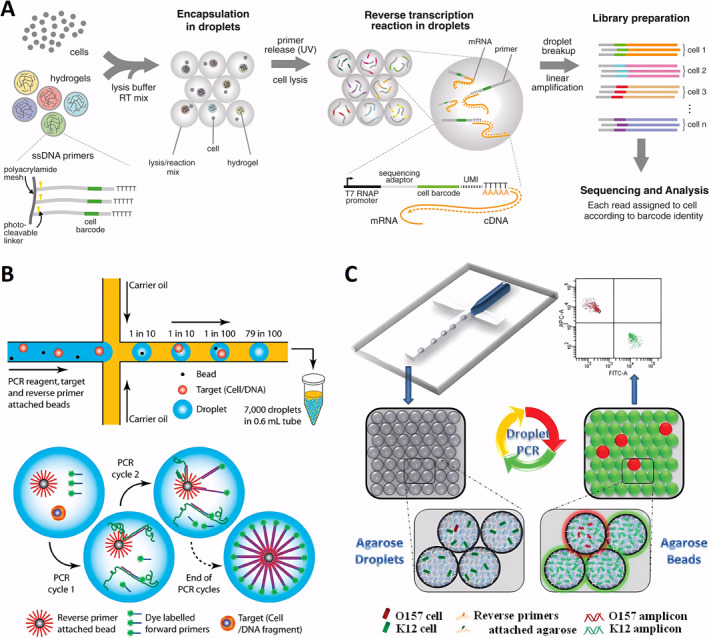
Droplet microfluidics for single‐cell genomic analysis. (A) The droplet microfluidics facilitate single‐cell transcriptomics by encapsulating cells with lysis buffer, barcoded primers, and hydrogel microspheres for efficient cDNA tagging during reverse transcription. Reproduced with permission [42]. Copyright 2015, Elsevier. (B) Individual cells and primer‐functionalized microbeads are encapsulated in PCR mix droplets, allowing for rapid amplification and analysis via flow cytometry. Reproduced with permission [43]. Copyright 2008, American Chemical Society. (C) Rare pathogen detection by agarose droplet microfluidic single‐cell ePCR technique. Reproduced with permission [44]. Copyright 2012, Royal Society of Chemistry.

Droplet microfluidics has revolutionized single‐cell transcript sequencing and driven the development of new platforms for single‐cell DNA and RNA sequencing. Since the introduction of droplet‐based highly parallelized single‐cell transcript sequencing by Weitz’s group in 2015 (Figure 6A), this technique has become the gold standard in the field [42]. It has fueled the recent surge in single‐cell data mining, offering unprecedented insights into a wide range of biological disciplines including developmental biology, tissue engineering, cancer biology, and microbiology. The ability to distinguish and quantify transcripts from thousands of individual cells within large populations has transformed our understanding of complex tissues and cellular functions. Genetic analysis of individual cells involves encapsulating single‐cells in droplets, lysing them to extract DNA, and performing subsequent amplification and detection. In a pioneering study, Kumaresan et al. utilized a droplet microfluidics generator to encapsulate single‐cell along with primer‐functionalized microbeads in an agarose solution that gels upon cooling (Figure 6B) [43]. The gel droplets were isolated from the oil phase, and a mixture of sodium dodecyl sulfate (SDS) and proteinase K was used to lyse the cells within the droplets. After lysis, interfering reagents were rinsed off, and a PCR mixture was added. The droplets were then re‐emulsified in oil, and thermocycling was performed to produce amplicon‐labeled beads, each carrying the genetic material of a single‐cell.

This high‐throughput single‐cell genetic analysis approach was validated through the successful analysis of the glyceraldehyde‐3‐phosphate dehydrogenase gene from human lymphocytes and the gyr B gene from bacterial E. coli K12 cells using rapid flow cytometry. Using a similar technique, the researchers analyzed mutant pathogenic cells within E. coli samples, identifying these mutants against a background of 10^5^ wild‐type cells. The use of agarose droplets instead of traditional aqueous droplets enhanced single‐genomic fidelity during cell lysis and DNA purification, and improved the efficiency of PCR emulsification. This approach enabled multilocus single‐cell sequencing of both the control gene β‐actin and the chromosomal translocation t(14; 18), a mutation present in 85%–90% of follicular lymphoma cases [45]. Precise pairing of individual cells with beads in a single droplet is critical in traditional methods, presenting a technical challenge. To address this, Yang et al. developed a microfluidic technique for emulsification PCR using bead‐free agarose droplets, with reverse primers covalently attached directly to the agarose. This approach increased droplet production efficiency by one to two orders of magnitude compared to conventional bead‐based systems. Agarose remains in a liquid state throughout all PCR cycling temperatures, effectively overcoming the limitations of solid surface PCR on beads, achieving a PCR efficiency of over 95%. Using this technique (Figure 6C), single‐cell PCR was successfully conducted on a background of 10^5^ excess normal K12 cells to identify a single pathogen, E. coli O15:H7 [44]. Additionally, the method was optimized for single‐cell RT‐PCR by injecting cells and RT‐PCR reagents from separate aqueous inlets. This setup allowed successful single‐cell RT‐PCR analysis, revealing significant differences in the expression levels of the cancer biomarker gene EpCAM across different cancer cell types at the single‐cell level [46]. This novel approach holds promise for applications in viral RNA detection and further single‐cell transcriptional studies, paving the way for breakthroughs in understanding gene expression heterogeneity.

## 7 Conclusion

In this study, we systematically summarize the significant advancements in droplet microfluidics for single‐cell analysis. These breakthroughs have made it possible to obtain high‐resolution data from individual cells, providing a broad prospect for exploring cellular activities. Despite the substantial progress made over the past decade, there remains a significant potential for further technological development, especially in terms of throughput and versatility. One of the primary challenges in droplet microfluidics is the limitation of throughput, mainly due to the small cross‐sectional area of the microchannels used for droplet generation, which restricts the speed of droplet formation. The current approach involves using multiple microchannels in parallel to generate droplets proportionally, which can achieve ultra‐high throughput to some extent. However, this still falls short of the ideal application levels, and further increasing throughput remains a key research goal for more rapid and efficient single‐cell analysis. To overcome throughput limitations, researchers are exploring various innovative technologies, such as developing new microchannel designs to optimize fluid flow paths, using advanced materials and manufacturing processes to reduce channel blockage and liquid retention, and integrating multi‐layer microchannel structures with automated control technologies. These improvements could significantly enhance the practical application of microfluidics in large‐scale single‐cell analysis. Although droplet microfluidics simplifies the cell analysis process by encapsulating individual cells within isolated droplets and significantly reduces the risk of cross‐contamination, it is still deficient in handling complex multi‐step processes. For example, procedures like sample mixing, washing, and multi‐stage reactions are not efficiently executed within droplet environments, which greatly limits the multifunctional development of microfluidic platforms. Currently, droplet microfluidics systems can primarily achieve basic functions such as cell encapsulation, incubation, fluorescence generation, and detection, but they struggle to handle more complex biochemical operations. This limitation affects their potential application in a wide range of biomedical research. To expand the functional modules of microfluidic systems, researchers are working on developing more flexible operational techniques, such as droplet fusion, division, manipulation, and multi‐step reaction integration.

Future research should focus on enhancing the versatility and integrated functions of microfluidic platforms, particularly in integrating sample processing, analysis, and data acquisition into a single microfluidic system. This could involve combining nanomaterials and biosensors for high‐sensitivity detection or designing intelligent microfluidic chips to achieve fully automated single‐cell analysis processes. Additionally, the introduction of artificial intelligence and machine learning technologies could offer new perspectives for data processing and analysis, pushing single‐cell research to a new level. With continuous technological improvements and functional expansion, droplet microfluidics is expected to overcome current bottlenecks and become a core tool in advancing frontier research in single‐cell analysis. This will not only deepen our understanding of cellular behaviors but also provide robust technical support for personalized medicine, disease diagnosis, and biopharmaceuticals.

## Author Contributions

Xiaoyu Xu conceived the topic of the manuscript, Chang Liu and Xiaoyu Xu wrote the manuscript.

## Conflicts of Interest

The authors declare no conflicts of interest.
